# What Are We Missing? SEEKing Expanded Support for Health-Related Social Needs

**DOI:** 10.1177/00099228251382047

**Published:** 2025-10-29

**Authors:** Ryan L. Spotts, Alana N. Snyder-Vyas, Chelsea Emrick, Kimberly Grey, Eric Schaefer, Howard Dubowitz, Benjamin N. Fogel

**Affiliations:** 1 Department of Pediatrics, Penn State College of Medicine, Hershey, PA, USA; 2 Cleveland Clinic, Cleveland, OH, USA; 3 University of Maryland School of Medicine, Baltimore, MD, USA

**Keywords:** social determinants of health, adolescent health, public health interventions, insurance disparities, family support services

## Abstract

Psychosocial screening is inconsistently administered in pediatric primary care and rarely includes older children. This cross-sectional study compares screening outcomes from and interventions related to expanded use of the safe environment for every kid (SEEK) approach in children aged 0 to 17 versus the usual application to families with children under 5. Caregivers (*N* = 450) of children aged 0 to 17 years completed screening between April and December 2021. Screening results were compared across 3 age categories (0-5, 6-11, and 12-17) using the exact binomial method and Fisher’s exact test. The prevalence of problems was similar across all age groups except for stress (*P* < .001) and substance misuse (*P* = .002). Publicly insured families had higher rates of identified problems versus privately insured families for stress (*P* = .035) and partner violence (*P* = .031). Interventions for positive screens were offered for 61% of participants. Expanded SEEK screening enables increased detection of social needs and offerings of resources for all ages and insurance types.

## Introduction

The World Health Organization defines social determinants of health (SDOH) as “the conditions in which people are born, grow, work, live, and age, and the wider set of forces and systems shaping the conditions of daily life.”^1^ Children living in stressful social and physical environments during key developmental periods can experience adversities affecting lifelong health and wellness.^1^ Repeated exposures to un-buffered toxic stress and adverse childhood experiences can lead to diseases in adulthood, maladaptive coping skills, unhealthy lifestyles, and fragmentation of social networks critical to health and success as people age.^2,3^ In acknowledgment of these potential harms, the American Academy of Pediatrics (AAP) provides guidance via a 2016 policy statement on the critical importance of screening for SDOH as part of pediatric care.^4^ Recognizing that each community may have unique and diverse needs, resources, and logistical considerations, the AAP does not specify which screener to use or how often to administer it.^4^ Developing a tailored approach to identify and mitigate problems for each patient population is paramount to supporting a healthier future for each community.

Despite the overwhelming support for pediatric primary care screening of SDOH, implementation is inconsistent with as few as 12% of pediatricians reporting the use of a standardized screening instrument.^1,5^ A systematic review of pediatric SDOH screening practices examined 17 studies using 11 different screening tools and found the majority of published data focuses on low-income urban populations and families with children under the age of 5.^1^ It is well-established that the majority of families irrespective of the ages of their children and socioeconomic status, experience psychosocial problems with over half of the US population experiencing at least one unmet social need.^6-8^ Thus, it is reasonable to evaluate more inclusive universal screening approaches for families with children of all ages and socioeconomic standings.

**Figure 1.**
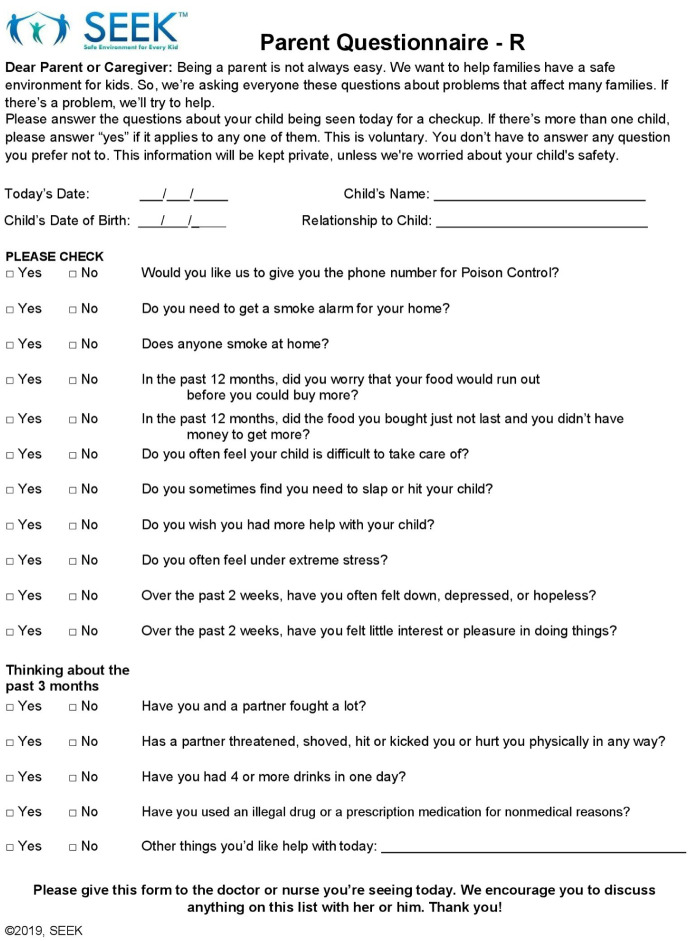
The safe environment for every kid (SEEK) parent questionnaire-revised (PQ-R).

The safe environment for every kid (SEEK) parent questionnaire-revised (PQ-R) is validated and commonly used SDOH screening tool administered to caregivers of children aged 0 to 5 years to briefly screen for problems associated with child maltreatment, adverse development, and safety (Figure 1).^9-15^ SEEK targets 6 key problems (food insecurity, harsh discipline, major stress, depression, intimate partner violence [IPV], and substance misuse) to assess possible stressors in the home environment that may impact health and wellness.^16-26^

Studies of the SEEK approach in families with children ages 0 to 5 years across varying socioeconomic status have demonstrated promising benefits to families, primary care professionals (PCPs), and health systems. Benefits to families include increases in resource provision to at-risk families and a reduction in child abuse, neglect, and harsh punishment. Benefits to PCPs include improved comfort and perceived competence while health systems benefit from an associated cost savings.^9-11,27^ Despite the wide acceptance and use of SEEK in pediatric primary care in 29 different states within the United States as well as in Sweden, limited application to only families with younger children may miss the opportunity to assist families with older children.^11^ Applying this same screening approach to caregivers of children ages 0 to 17 years will test the hypothesis that the targeted problems are still prevalent in families with older children. The inclusive application of expanded screening approaches to economically diverse populations will additionally test the hypothesis that not only targeted problems are present in financially disadvantaged families but also identifying these issues in all socioeconomic groups leads to increased offerings of services tailored to individual patient population needs.

## Study Design

This study involved a convenience sample of families bringing their children to pediatric well-child visits. Due to the fact that all SEEK questions are dichotomous and most of the answers were expected to be skewed to the choice of “no,” a relatively large sample was needed to generate meaningful results.^28^ Therefore, this study targeted the inclusion of approximately 150 participants from 3 different age groups (0-5, 6-11, and 12-17) yielding a total sample size of about 450. To enhance the diversity of the sample, participants were randomly selected in equal portions from 3 suburban academic general pediatric offices and participating PCPs (*N* = 21).

During the April to December 2021 study period, there were 13 697 well visits performed across all practice sites where parents and caregivers were given the SEEK PQ-R. Potential participants were generally white (65%), non-Hispanic (77%), and privately insured (62%). Parents and caregivers were directed by office staff to complete paper questionnaires in the waiting or exam rooms prior to meeting with their PCP.

Participating PCPs were all asked to complete the online training for the SEEK approach to prepare them to briefly address identified problems, by utilizing motivational interviewing principles, and through the use of response to barriers algorithms designed to help overcome obstacles to the acceptance to care.^29^ A pediatric social worker equipped clinics with SEEK parental handouts customized with local resources for each of the targeted problems. Primary care professionals were instructed to review the responses with the families, discuss available resources, including counseling or reassurance, handouts, and referrals, and document any interventions within the electronic health record (EHR). Parents and caregivers were offered the opportunity to speak with the social worker if they requested additional resources beyond the SEEK handouts or their results revealed more complex social needs. Primary care professionals and social workers respected the caregiver’s self-determination if they declined additional resources. The paper SEEK PQ-R form was scanned into the EHR allowing retrospective chart review of screening results and associated PCP documentation of any interventions offered. At the end of the 9-month study period, de-identified aggregate data were reviewed from 450 charts selected via a random number generator from a representative sample of PCPs from each practice site, including subsamples of 151 (33.6%) patient charts from the age group of 0 to 5 years, 173 (38.4%) from the age group of 6 to 11 years, and 126 (28%) from the age group of 12 to 17 years. Only patients with a scanned SEEK PQ-R within the EHR were included in the chart review and analysis. Problems identified and the types of interventions offered were recorded.

## Statistical Methods

Each of the 6 problems targeted by SEEK has 2 associated questions; an affirmative response to either or both indicates a positive screen. The rates of positive responses for each targeted problem were described for the entire sample and stratified by age groups (0-5, 6-11, and 12-17). Proportions of positive response between groups were compared using Fisher’s exact test. For those targeted problems with significant differences between age groups, we conducted follow-up pairwise comparisons using Fisher’s exact test. The exact binomial method was used to calculate confidence intervals for the prevalence of each problem. Fisher’s exact test was also used to compare the relationships between insurance type, serving as a proxy for socioeconomic status, and both the age groups of participants and the positivity rates of screenings, as well as the interventions offered by PCPs. This study was approved by the Penn State Institutional Review Board.

## Results

A total of 450 randomly selected charts with completed SEEK PQ-R were reviewed over the 9-month study period for pediatric well visits aged 0 to 17 years. Participants were primarily white (51.1%), non-Hispanic (79.6%), and privately insured (57.0%) (see Table 1). As age increased, the proportion of participants with public insurance decreased from 52% for ages 0 to 5 to 41% for ages 6 to 11, and 32% for ages 12 to 17 (*P* = .023). The parent or caregiver who completed the form was most often the mother (*n* = 324, 72.0%).

**Table 1.** Demographics of the Children and Their Caregivers (*N* = 450).

	Age, 0-5 (*n* = 151)	Age, 6-11 (*n* = 173)	Age, 12-17 (*n* = 126)	Total (*N* = 450)
**Race**				
White	78 (51.7%)	92 (53.2%)	60 (47.6%)	230 (51.1%)
African American	16 (10.6%)	11 (6.4%)	20 (15.9%)	47 (10.4%)
Asian	13 (8.6%)	14 (8.1%)	3 (2.4%)	30 (6.7%)
Mixed	15 (9.9%)	25 (14.5%)	22 (17.5%)	62 (13.8%)
Not available	29 (19.2%)	31 (17.9%)	21 (16.7%)	81 (18.0%)
**Ethnicity**				
Hispanic	14 (9.3%)	10 (5.8%)	8 (6.3%)	32 (7.1%)
Not Hispanic	120 (79.5%)	139 (80.3%)	99 (78.6%)	358 (79.6%)
Not available	17 (11.3%)	24 (13.9%)	19 (15.1%)	60 (13.3%)
**Gender**				
Male	74 (49.3%)	91 (52.6%)	51 (40.5%)	216 (48.1%)
Female	76 (50.7%)	82 (47.4%)	75 (59.5%)	233 (51.9%)
Not available	1 (< 1.0%)	0	0	1 (<1.0%)
**Insurance** ^a^				
Private	72 (47.7%)	98 (57%)	86 (68.3%)	256 (57.0%)
Public	78 (51.7%)	71 (41.3%)	40 (31.7%)	189 (42.1%)
No insurance	1 (< 1.0%)	2 (1.2%)	0	3 (< 1.0%)
Not available	0	2 (< 1.0%)	0	2 (< 1.0%)
**Relationship**				
Mother	110 (72.8%)	126 (72.8%)	88 (69.8%)	324 (72.0%)
Father	26 (17.2%)	24 (13.9%)	18 (14.3%)	68 (15.1%)
Legal guardian	2 (1.3%)	1 (< 1.0%)	0	3 (< 1.0%)
Unknown	13 (8.6%)	22 (12.7%)	20 (15.9%)	55 (12.2%)

a Insurance status was significantly (*P* = .023) different between groups. No significant difference among other characteristics.

The positive response rate across the entire sample (all age groups) was relatively low (1%-10%) for each problem. The highest positive response rate was observed for major stress (9.8%) while the lowest rates were for substance misuse (1.1%) and food insecurity (1.1%). A total of 57% of positive screens came from parents or caregivers of children aged 6 to 17 years. Table 2 compares positive response rates in the 3 age groups; significant differences occurred only for major stress (*P* < .001) and substance misuse (*P* = .002). Within these targeted problems, follow-up tests for each age comparison showed that the percentage of participants with major stress was significantly lower for ages 6 to 11 years compared with ages 0 to 5 years (4% vs 17%; *P* < .001) and compared with ages 12 to 17 years (4% vs 10%; *P* = .028). For substance misuse, rates for ages 0 to 5 and 6 to 11 years were significantly lower than 12 to 17 years (0% vs 4% for both; *P* = .02 for both).

**Table 2.** Responses for Each SEEK Targeted Problem, by Age Group.

	Age, 0-5 (*n* = 151)	Age, 6-11 (*n* = 173)	Age, 12-17 (*n* = 126)	Total (*N* = 450)	*P* value
**Food insecurity**					.63
Yes	2 (1.3%)	1 (0.6%)	2 (1.6%)	5 (1.1%)	
95% CI	(0.2, 4.7)	(0.0, 3.2)	(0.2, 5.6)	(0.4, 2.6)	
**Harsh punishment**					.19
Yes	1 (0.7%)	6 (3.5%)	4 (3.2%)	11 (2.4%)	
95% CI	(0.0, 3.6)	(1.3, 7.4)	(0.9, 7.9)	(1.2, 4.3)	
**Major stress**					< .001
Yes	25 (16.6%)	6 (3.5%)	13 (10.3%)	44 (9.8%)	
95% CI	(11.0, 23.5)	(1.3, 7.4)	(5.6, 17.0)	(7.2, 12.9)	
**Depression**					.08
Yes	14 (9.3%)	6 (3.5%)	6 (4.8%)	26 (5.8%)	
Missing	0	1	0	1	
95% CI	(5.2, 15.1)	(1.3, 7.4)	(1.8, 10.1)	(3.8, 8.4)	
**Intimate partner violence**					.33
Yes	1 (0.7%)	5 (2.9%)	3 (2.4%)	9 (2.0%)	
Missing	2	1	1	4	
95% CI	(0.0, 3.7)	(1.0, 6.7)	(0.5, 6.9)	(0.9, 3.8)	
**Substance misuse**					.002
Yes	0	0	5 (4.0%)	5 (1.1%)	
Missing	3	1	0	4	
95% CI	(0.0, 2.5)	(0.0, 2.1)	(1.3, 9.0)	(3.6, 2.6)	

Individual problem rates were then analyzed by stratifying by insurance type as a proxy for socioeconomic status to compare its relationship with problem prevalence (see Table 3). Families utilizing public insurance had higher rates of identified problems than those insured privately for major stress (13.2% vs 7%, *P* = .035) and IPV (3.7% vs 0.8%, *P* = .031). Other targeted problems had no significant difference by insurance type.

**Table 3.** Responses for Each SEEK-Targeted Problem, by Insurance Type.

	Private (*n* = 256)	Public (*n* = 189)	*P* value
**Food insecurity**			.65
Yes	2 (0.8%)	3 (1.6%)	
95% CI	(0.1, 2.8)	(0.3, 4.6)	
**Harsh punishment**			.34
Yes	4 (1.6%)	6 (3.2%)	
95% CI	(0.4, 4.0)	(1.2, 6.8)	
**Major stress**			.035
Yes	18 (7%)	25 (13.2%)	
95% CI	(4.2, 10.9)	(8.7, 18.9)	
**Depression**			.06
Yes	10 (3.9%)	16 (8.5%)	
Missing	0	1	
95% CI	(1.9, 7.1)	(4.9, 13.5)	
**Intimate partner violence**			.031
Yes	2 (0.8%)	7 (3.7%)	
Missing	3	1	
95% CI	(0.1, 2.8)	(1.5, 7.5)	
**Substance misuse**			.99
Yes	3 (1.2%)	2 (1.1%)	
Missing	3	1	
95% CI	(0.2, 3.4)	(0.1, 3.8)	

Four patients were excluded from this analysis for missing insurance information.

Inclusive of all age participants, PCPs documented offerings of interventions at varying rates dependent upon the targeted problem (see Table 4). The most commonly offered intervention involved counseling or reassurance (20%-34%). Other interventions included handout provision (0%-40%), social work referrals (0%-15%), and community agency referrals (0%-11%). There were no differences in offerings of interventions by PCPs related to type of insurance.

**Table 4.** Interventions Offered for Identified Problems and Insurance Type, for All Age Groups.

	Private	Public	Total	p-Value
**Food insecurity positive**	*n* = 2	*n* = 3	(*N* = 5)	1.0
Yes intervention	2 (100%)	2 (66.7%)	4 (80%)	
**Harsh discipline positive**	*n* = 4	*n* = 6	(*N* =10)	0.08
Yes intervention	0 (0%)	4 (66.7%)	4 (40%)	
**Major stress positive**	*n* = 18	*n* = 25	(*N*=43)	1.0
Yes intervention	11 (61%)	16 (64%)	28 (63%)	
**Depression positive**	*n* = 10	*n* = 16	(*N*=26)	0.43
Yes intervention	7 (70%)	8 (50%)	15 (57.7%)	
**Intimate partner violence positive**	*n* = 2	*n* = 7	(*N* = 9)	1.0
Yes intervention	2 (100%)	5 (71%)	7 (77.8%)	
**Substance misuse positive**	*n* = 3	*n* = 2	(*N* = 5)	1.0
Yes intervention	1 (33.3%)	1 (50%)	2 (40%)	

Four patients were excluded from this analysis for missing insurance information.

## Discussion

In this initial pilot study of the expansion of SEEK screening for SDOH to families with older children, we found that SEEK identifies health-related social stressors in all families irrespective of the age or socioeconomic status of the child. Problem rates for families with children 5 and under for this study were similar when compared with published data from other institutions using SEEK screening (food insecurity 1.3% vs 4%, harsh discipline ~1% vs 3%, major stress 16.5% vs 7%, parental depression 9% vs 5%, substance misuse 0% vs < 1%, IPV 1% vs 1%) within a demographically similar population (94.3% white, 30% public insurance).^30^ The overall low screening positivity rates of this study highlight probable parental barriers to disclosing socially sensitive problems to their child’s PCP. For example, according to 2021 national and local statistics, we could expect approximately 10% to 15% of families to be experiencing food insecurity compared with the 1.1% of positive responses within this study.^31,32^ Similar discrepancies are seen when comparing parental depression (5.8% vs 28.5%), major stress or anxiety (9.8% vs 42.7%), harsh discipline (2.4% vs 52.5%), IPV (2% vs 14%-25%), and substance misuse (1.1% vs16.5%) rates within this study to other published data.^33-36^ These differences are unlikely to represent lower true problem rates within the study population. Rather, they highlight probable barriers to disclosing information associated with evoking emotions, such as shame, guilt, or embarrassment, the lack of a trusting relationship between caregivers and PCPs, or simply the medium by which the screening was administered (paper vs digital).^37-39^

When comparing the responses from caregivers of the 3 age groups, there were similar positivity rates for all problems except for major stress (*P* < .001) and substance misuse (*P* = .002). Consequently, health systems currently restricting screening to only children aged 5 years and under may anticipate that expanding their screening programs to include older children would yield positive disclosure rates from parents or caregivers with older children at similar frequencies to what they are seeing in their younger population. It is not surprising that families with older children often face similar challenges to those with younger ones. For example, it is well-established that adolescence can be a particularly difficult time for parents and teens.^40^

Differences in problem prevalence in SDOH screening approaches would be expected secondary to variance in social advantages experienced by each population.^41^ Using insurance type as a proxy for socioeconomic status, higher problem rates within this study were observed for major stress and IPV in lower-income families. This underscores how populations with fewer resources are often disproportionately burdened with more stressful environments; they may stand to experience even greater benefits from expanded SDOH screening approaches. Despite these differences, it is crucial to understand that there were psychosocial problems disclosed by parents irrespective of socioeconomic status in all areas targeted by SEEK.

It is also important to recognize that expanded screening should be directly linked to offerings of assistance as it is axiomatic that screening should lead to potential benefits for the participating population. Within this study, PCPs sub-optimally documented (61%) offerings of help following positive screens on average for all problems targeted by SEEK. In addition, there was a wide variance between these offerings relative to the disclosure of each specific problem (40%-80%). The overall low rate of response may be best explained by PCPs simply underreporting the complete details of any help they may have offered. It may also demonstrate additional barriers, such as inadequate training to address problems or a low confidence in the effectiveness of available resources.^42^ The variance in PCP response relative to the disclosed caregiver problem is likely more complicated. It is likely that inconsistencies in PCP comfort exist related to the perceived sensitivity or associated stigma of any given problem. For example, a PCP was twice as likely to offer help for food insecurity when compared with substance misuse (80% vs 40%). There may also be differences in response related to the PCP’s perception for immediate risk. For example, PCPs responded to concerns related to IPV at a higher rate when compared with caregiver’s use of harsh discipline (77.8% vs 40%). Primary care professionals may also respond in varying degrees due to recognition of inadequate skills or simply insufficient time for problems they deem to be more labor intensive. Notably, there were no statistical differences in PCP offerings of interventions within this study related to insurance type, but conclusive statements are limited secondary to small and underpowered sample.

Justification for the expansion of a screening approach with overall low detection and, at times, low PCP response rates may be debated. However, any identification of concerns and resultant PCP led offerings of assistance for infrequent problems that can seriously impair children’s health, safety, development, and wellbeing, strongly supports both the continuation and expansion of the SEEK approach.^9^ This calls for perhaps improving the SEEK approach, including better training of PCPs and monitoring of its implementation.

## Limitations

The clinics participating in this study were located in largely rural and suburban areas, with a demographically different patient population than that in most studies examining SDOH screening in pediatrics.^1^ It is likely that more socially disadvantaged and urban populations would have different rates of positive screens. In addition, the random selection of 450 participants only represents a small percentage (3%) of the total eligible clinic population and sampling biases of patients with more or fewer problems are possible. Every reasonable effort was made to prioritize the confidentiality of participant responses. However, caregiver perceptions of available privacy and resultant response truthfulness may have varied secondary to the method of screener administration (paper vs electronic forms), location (waiting room vs exam room) of form completion, and awareness of private health information documentation availability within online portal systems. Intervention data may have been limited secondary to variance among PCP documentation practices and adherence to standardized well visit templates. It is likely that PCPs under-documented their offerings of assistance and underreported any interventions discussed.

## Conclusion

Expansion of the SEEK approach to all pediatric age groups enables PCPs to help families with older children that would otherwise be missed by traditional age-limited SEEK screening practices. These promising findings help support a need for the broader application of SEEK to benefit more families. Additional studies examining the SEEK approach in families with older children as part of demographically different patient populations and its impacts on parenting, strength of families, child health, development, family wellbeing, and rates of child maltreatment, are still needed.

## Author Contributions

Drs Ryan Spotts, Alana Snyder, Howard Dubowitz, and Benjamin Fogel conceptualized and designed the study, drafted the initial manuscript, and critically reviewed and revised the manuscript.

Chelsea Emrick and Kimberly Grey conceptualized and designed the study, coordinated and supervised data collection, and critically reviewed and revised the manuscript.

Eric Schaefer conceptualized and designed the study, carried out the initial analyses, and critically reviewed and revised the manuscript.
